# Surgical repair of a traumatic carotid-jugular arteriovenous fistula

**DOI:** 10.1590/1677-5449.200008

**Published:** 2020-11-16

**Authors:** Adenauer Marinho de Oliveira Góes, Salim Abdon Haber Jeha, Daniela Vale Dias, Juliana Medley Torres Ferreira

**Affiliations:** 1 Universidade Federal do Pará (UFPA), Belém, PA, Brasil.

**Keywords:** arteriovenous fistula, wounds and injuries, jugular vein, carotid artery

## Abstract

Penetrating neck injuries can be life threatening. In about 25% of cases there are vascular injuries, which can progress to formation of arteriovenous fistulas. The authors present a case of delayed open surgery to repair a carotid-jugular fistula and briefly review the diagnosis and treatment options for this condition.

## INTRODUCTION

Some arterial injuries are complicated by the development of arteriovenous fistulas (AVFs), which occur as abnormal communications between arteries and veins caused by iatrogenic or traumatic injuries.^1^^,^^2^ A penetrating trauma can lead to formation of pseudoaneurysms (PSA) and, if there are also venous injuries, to formation of an AVF.^2^^,^^3^ If left untreated, depending on their site and size, these vascular injuries can cause complications such as congestive heart failure, cerebral ischemia, thromboembolism, or bleeding.^3^^,^^4^

The first observations of AVFs were made by Hunter in 1757 and published in 1764.^5^ The first description of a post-traumatic carotid-jugular fistula was published by Warren et al. in 1951. They studied cardiac output in people with AVFs who had been wounded in the Second World War.^6^

Arteriovenous fistulas should be repaired as soon as possible. Surgery is less difficult during the initial stages, because fibrosis and collateral circulation distort the anatomy and increase the risk of dissection.^3^^,^^7^ Early intervention can also prevent hemorrhages and formation of PSA. These injuries can be treated using conventional surgical techniques or endovascular methods.^4^^,^^7^^,^^8^

## PART I – CLINICAL SITUATION

The patient was a 39-year-old man, victim of gunshot wounds to the neck and left forearm 8 months before the occurrence. He was treated at a public hospital, where he was admitted and underwent surgery to treat fractured bones in his forearm, but was discharged without investigation of vascular injuries.

The patient complained of a constant “buzzing” sound and a swelling in the neck, which he had noticed soon after discharge, and had been worsening gradually. When interviewed, the patient reported that he had a low income and lived a long way from any large urban center, but did not describe any other relevant history.

**Figure 1:**
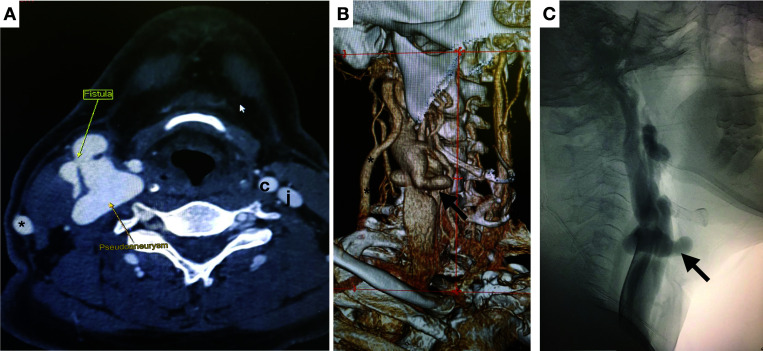
Preoperative examinations. **A**) Computed tomography of the neck (axial slice); **B**) volumetric rendering; **C**) selective arteriography of the right common carotid (lateral view). Smaller arrow: arteriovenous fistula; larger arrow: carotid pseudoaneurysm; * right external jugular; c: left common carotid, j: left internal jugular.

On physical examination, the scar caused by a through-and-through gunshot wound to right cervical zone II was identified and a large pulsating mass with intense murmur and thrill was found. The initial diagnostic hypothesis was traumatic AVF. Angiotomography (angioCT) confirmed the presence of a carotid-jugular AVF and revealed a carotid PSA. The patient underwent a selective arteriography which confirmed a high-output fistula, with no other findings beyond those of the angioCT (Figure 1).

The options considered were endovascular repair of the fistula with placement of a covered stent, or conventional surgical repair with a synthetic or autologous vein graft.

## PART II – WHAT WAS DONE

**Figure 2:**
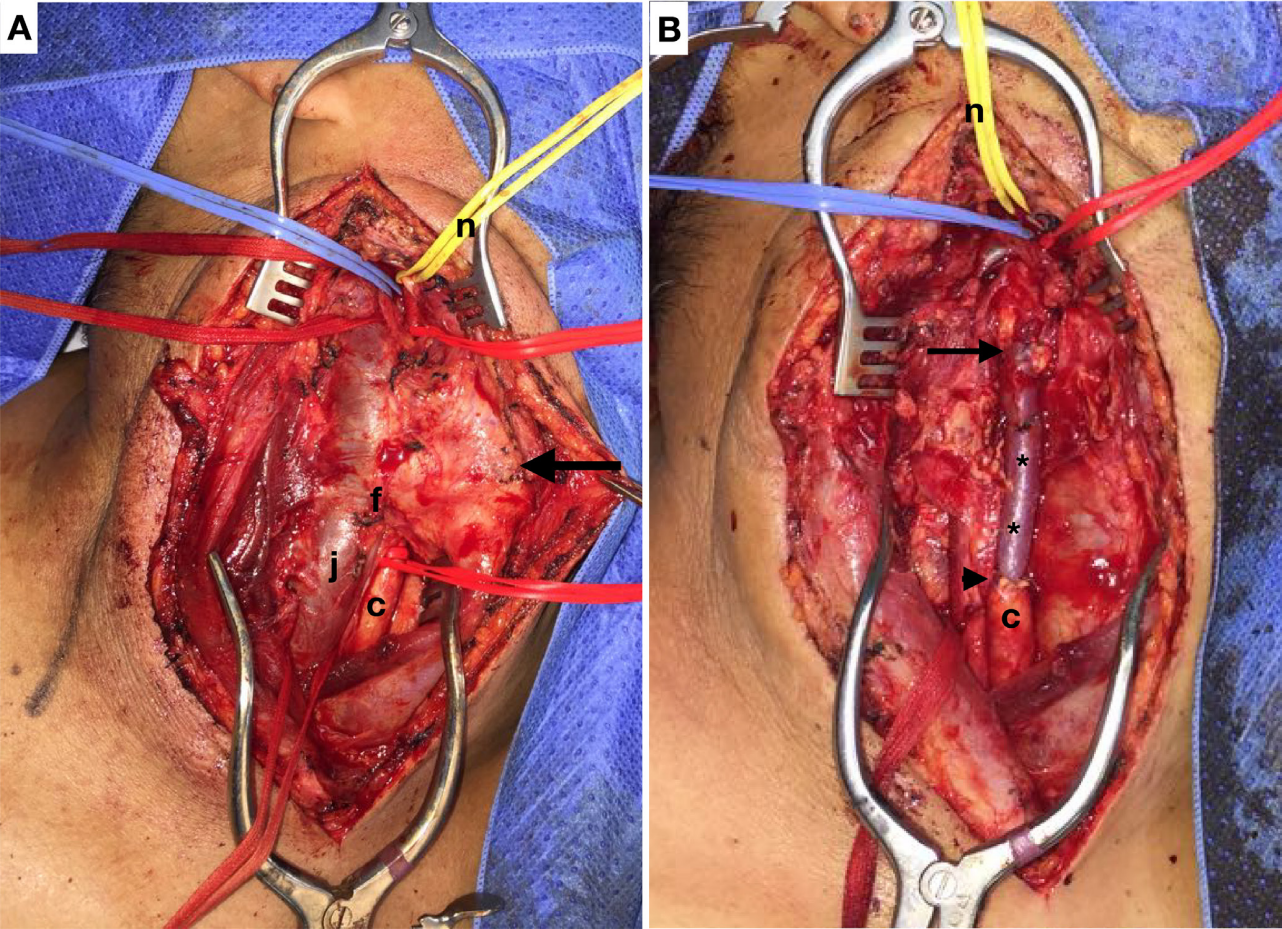
Intraoperative images. **A**) Before repair of the injury and **B**) after repair. Larger arrow: carotid pseudoaneurysm; f: arteriovenous fistula; * right external jugular vein graft; c: right common carotid, j: right internal jugular; n: repair of hypoglossal nerve.

The treatment chosen was open repair. Surgery was performed under general anesthesia, via a cervicotomy incision following the anterior margin of the right sternocleidomastoid muscle. During dieresis, the external jugular vein was resected and maintained in a heparin solution, the AVF and the carotid PSA were identified and proximal and distal arterial and venous flow were controlled (it was necessary to isolate the internal and external carotids to achieve distal arterial control) (Figure 2).

**Figure 3:**
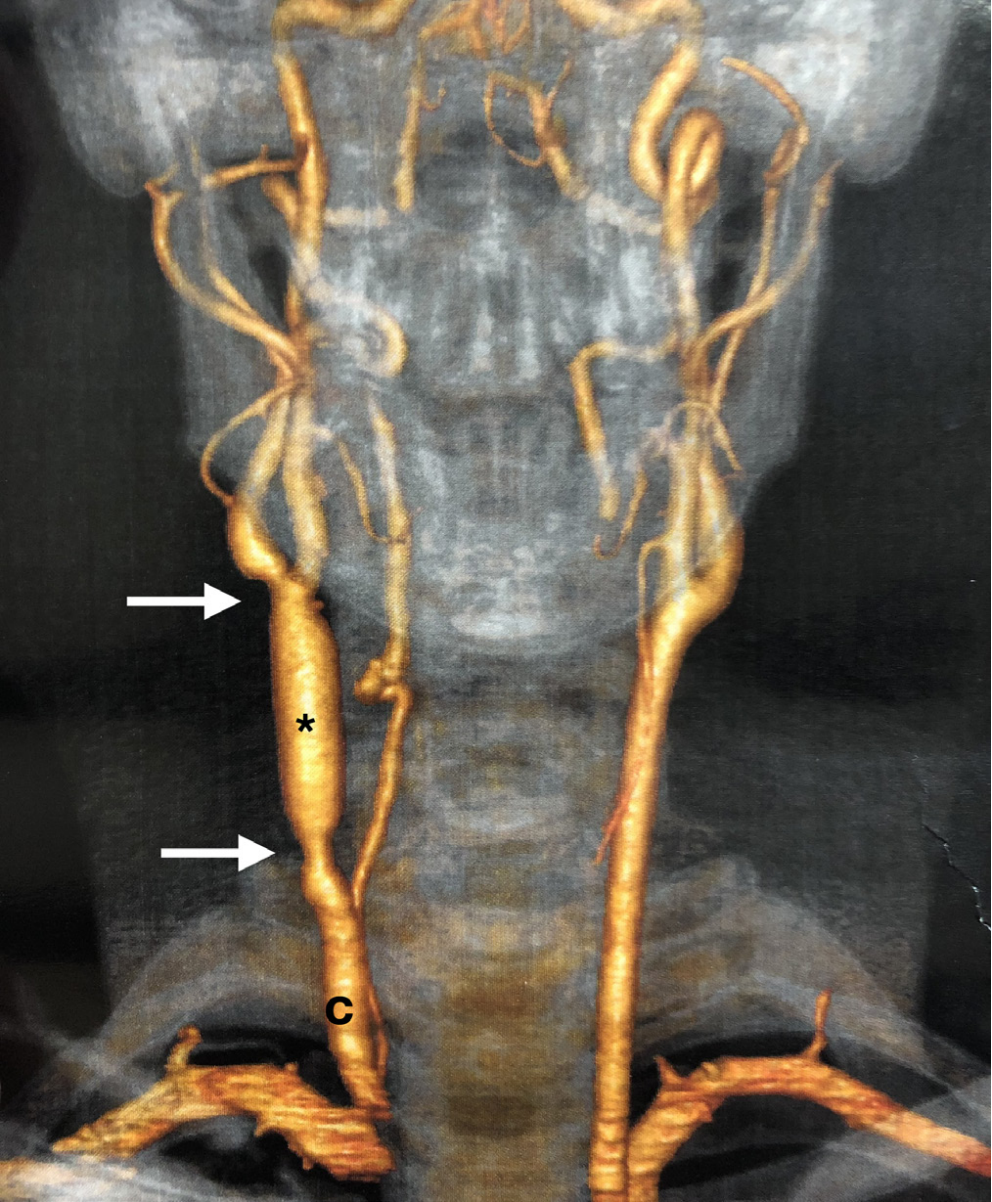
Computed tomography of the neck (volumetric rendering) in 18th postoperative month. * right external jugular vein graft; c: right common carotid, the arrows indicate the proximal and distal anastomoses.

After control of vascular flow had been achieved, the patient was given systemic anticoagulation and arterial and venous occlusions were accomplished proximal to and distal of the injury. A longitudinal venotomy revealed a large parietal defect allowing communication with the arterial lumen. The damaged section was resected with ligature of the venous stumps, and the external jugular graft that had been harvested earlier was used to repair the carotid with end-to-end anastomoses, with the distal anastomosis at the level of the carotid bifurcation (Figure 3).

A negative-pressure drain was fitted, exiting via a counter incision and left in place for 24 hours. The patient was discharged from hospital on the second postoperative day and was instructed to take 100 mg/day of acetylsalicylic acid for 3 months and to attend for regular follow-up consultations.

The case has been followed-up for 18 months, with no neurological complications. A recent control angioCT showed that the venous graft was patent, but also revealed discrete dilation increasing its caliber along the entire length of the graft. Color Doppler ultrasonography showed that the carotid system was patent, with laminar flow through the internal carotid and in the common carotid upstream of the anastomosis. However, flow through the graft was turbulent, because of its uniform dilation, although without hemodynamic repercussions. Peak systolic velocity recorded was 87.9 cm/s in the common carotid upstream of the graft and 69.2 cm/s in the internal carotid.

## DISCUSSION

The neck is vulnerable to injuries that can involve blood vessels, nerves, the trachea, and the esophagus.^9^ Approximately 25% of penetrating traumas to the neck cause vascular injuries^7^ and carotid injuries are potentially fatal, due to hemorrhage, airway compression, and stroke.^7^^,^^10^ Carotid-jugular fistulas are not frequent, accounting for just 4% of all traumatic AVFs.^11^

Diagnosis of traumatic AVF and PSA requires careful history taking and clinical examination, although noninvasive and invasive diagnostic methods may also be needed.^4^^,^^12^ Signs and symptoms of AVF include pulsation in the neck area, edema, systolic murmur, thrill, and dilation of superficial veins.^12^^-^14 A machinery (or locomotive) murmur is pathognomonic for AVF^15^ and the Nicoladoni-Branham sign, comprising bradycardia and increased mean blood pressure in response to manual compression of the fistula, can be observed in some cases.^16^^-^18

Immediate surgical intervention is indicated in cases with evident vascular injury and hemodynamic instability.^8^ Elective treatments should be preceded by careful therapeutic planning, including anatomic assessment of the structures involved.^4^^,^^12^ AngioCT, magnetic resonance imaging (MRI), and Doppler ultrasonography are often employed and, in some cases, angiography can play an important role in planning, whether for conventional, endovascular, or hybrid surgery.^4^^,^^16^^,^^18^ In the case described above, arteriography only showed intracranial opacification when the catheter was advanced distally of the AVF. When it was in a proximal position, all of the flow was diverted to the venous system via the AVF, demonstrating that, from a functional point of view, the effect was equivalent to carotid ligature, which is why surgical reconstruction was possible without a vascular shunt.

In cases with anatomic distortion, such as in large AVFs or when there is also a PSA, endovascular techniques offer advantages over surgical dissection of the structures involved.^7^^,^^12^ The choice of technique should take account of anatomic characteristics, the size of the aneurysm sac, the degree of arterial injury, and distal arterial flow.^7^^,^^14^ Additionally, availability of materials, the age group of the patient, and the conditions for long-term follow-up should also be considered.

Endovascular techniques are frequently indicated for vertebral artery injuries involving the carotids in zones I or III, where surgical access is more difficult.^7^^,^^18^ The advantages of this approach are a less invasive remote access, less hemorrhage, shorter length of hospital stay, and, consequently, lower cost.^7^^,^^12^ However, studies have shown that endovascular treatment of traumatic AVFs and PSAs involves a potential risk of late intra-stent stenosis, thromboembolism, and formation of PSA at the site of arterial puncture.^4^^,^^12^

With regard to conventional surgical techniques, open repair of a carotid-jugular fistula can be accomplished using grafts, end-to-end anastomoses, (patches), sutures, and ligatures;^8^ although ligature should be avoided when possible because it is associated with less favorable results than arterial reconstruction.^9^ Additionally, arterial reconstruction with autologous vein grafts is a more durable solution with a lower risk of infection than prosthetic materials.^3^^,^^4^ However, venous grafts may undergo late dilation, especially in younger patients, as in the case presented here, demanding careful follow-up.^2^^,^^3^

The great saphenous vein can be used, with low rates of thrombosis and infection, but this requires an additional incision in the lower limb, with consequent increase in the duration of surgery.^2^^,^^8^ Considering the benefits of an autologous graft, and since the external jugular vein was already dilated, with thickening of the wall (because of pressurization by the AVF), the surgical team decided to use it, avoiding the need for additional incisions and reducing the duration of the operation.

Endovascular treatment was available and placement of a covered stent would have been feasible and technically simpler than surgical dissection in a case such as this one. However, the conventional technique was chosen instead of an endovascular approach because of the risks of late complications and the lack of studies investigating its long-term efficacy, since the patient was very young.^2^ Furthermore, endovascular repair would also have needed long-term postoperative antiplatelet treatment to avoid thromboembolic complications and occlusion of the stent and also follow-up with regular imaging exams, which would have made adhesion to treatment less likely, especially since the patient was in a situation of socioeconomic vulnerability.^4^^,^^19^
