# Supplementary material for: Surgical repair of a traumatic carotid-jugular arteriovenous fistula
Source: J Vasc Bras. 2020 Nov 16;19:e20200008. doi: 10.1590/1677-5449.200008 (PMC8218008; doi:10.1590/1677-5449.200008)
Supplement: Translation

# Translation: Correção cirúrgica de fístula carotídeo-jugular traumática

**Authors:** Adenauer Marinho de Oliveira Góes, Salim Abdon Haber Jeha, Daniela Vale Dias, Juliana Medley Torres Ferreira

**Affiliations:** 1 Universidade Federal do Pará (UFPA), Belém, PA, Brasil.

**Keywords:** fístula arteriovenosa, ferimentos e lesões, veia jugular, artéria carótida

## Abstract

Traumatismos cervicais penetrantes podem ser potencialmente fatais. Em cerca de 25% dos casos, há lesão vascular associada, que pode evoluir para fístulas arteriovenosas. Os autores apresentam um caso de correção tardia, por cirurgia aberta, de uma fístula carotídeo-jugular e fazem uma breve revisão sobre o diagnóstico e opções de tratamento dessa condição.

## INTRODUÇÃO

As lesões arteriais podem ser complicadas pelo desenvolvimento de fístulas arteriovenosas (FAVs), que ocorrem como comunicações anormais entre artérias e veias devido a lesões iatrogênicas ou traumáticas^1^^,^^2^. Um trauma penetrante pode levar à formação de pseudoaneurismas (PSA) e, se houver lesão venosa concomitante, ao desenvolvimento de FAV^2^^,^^3^. Se não tratada, dependendo de sua localização e tamanho, essa lesão vascular pode apresentar complicações como insuficiência cardíaca congestiva, isquemia cerebral, tromboembolismo ou sangramento^3^^,^^4^.

As primeiras observações de FAVs foram feitas por Hunter em 1757 e publicadas em 1764^5^. A primeira descrição de fístula carotídeo-jugular pós-traumática foi publicada por Warren et al. em 1951. Esses autores estudaram o débito cardíaco em portadores de FAVs feridos durante a Segunda Guerra Mundial^6^.

As FAVs devem ser corrigidas assim que possível. Nos estágios iniciais, a cirurgia é mais fácil, pois a fibrose e a circulação colateral distorcem a anatomia e aumentam o risco da dissecção^3^^,^^7^. Além disso, a intervenção precoce pode prevenir hemorragias e a formação de PSA. O tratamento dessas lesões pode incluir tanto técnicas de cirurgia convencional quanto endovasculares^4^^,^^7^^,^^8^.

## PARTE I – SITUAÇÃO CLÍNICA

Um homem de 39 anos apresentava história de ferimentos por projéteis de arma de fogo (FPAF) no pescoço e no antebraço esquerdo há 8 meses. Foi atendido em um hospital público, internado e submetido a tratamento cirúrgico por fratura de ossos do antebraço. Posteriormente, recebeu alta sem que lesões vasculares tivessem sido investigadas.

O paciente referiu “zumbido” constante e aumento do volume cervical, que iniciaram pouco tempo após a alta e pioraram de modo insidioso. Na anamnese, detectou-se ainda que era de baixa renda e morava em localidade afastada de grandes centros urbanos. Não havia antecedentes relevantes.

Ao exame físico, observava-se cicatriz resultante do ferimento transfixante na zona cervical II direita, uma volumosa massa pulsátil com sopro e frêmito intensos. A hipótese diagnóstica inicial foi de FAV traumática. Uma angiotomografia (angioTC) confirmou a presença de FAV carotídeo-jugular e demonstrou PSA de carótida. O paciente foi submetido a uma arteriografia seletiva que confirmou o alto débito da fístula, sem outros achados em relação à angioTC (Figura 1).

**Figura 1:** Exames pré-operatórios. **A**) Tomografia computadorizada do pescoço (corte axial); **B**) renderização volumétrica; **C**) arteriografia seletiva da carótida comum direita (perfil). Seta menor: fístula arteriovenosa; seta maior: pseudoaneurisma carotídeo; * jugular externa direita; c: carótida comum esquerda, j: jugular interna esquerda.

Cogitou-se, então, a correção da fístula por meio endovascular, implantando-se um stent revestido, ou por meio da cirurgia convencional, utilizando-se enxerto sintético ou com veia autóloga.

## PARTE II – O QUE FOI FEITO

Optou-se por correção aberta e o procedimento foi realizado sob anestesia geral, através de cervicotomia acompanhando a borda anterior do músculo esternocleidomastoideo direito. Durante a diérese, a veia jugular externa foi ressecada e mantida em solução com heparina, foi identificada a área da FAV e do PSA carotídeo e foram obtidos controles proximais e distais tanto arteriais quanto venosos (para o controle arterial distal, foi necessário isolar as carótidas interna e externa) (Figura 2).

**Figura 2:** Imagens do ato cirúrgico. **A**) Antes da correção da lesão e **B**) após. Seta maior: pseudoaneurisma carotídeo; f: fístula arteriovenosa; * enxerto com jugular externa direita; c: carótida comum direita, j: jugular interna direita; n: reparo do nervo hipoglosso.

Após a obtenção dos controles vasculares, o paciente foi submetido a anticoagulação sistêmica e oclusões arteriais e venosas proximais e distais à lesão. Uma venotomia longitudinal permitiu identificar o grande defeito parietal com comunicação para o lúmen arterial. Optou-se pela ressecção do segmento lesionado, com ligadura dos cotos venosos, e a reconstrução carotídea foi realizada com enxerto da jugular externa previamente colhida, feitas anastomoses termino-terminais, sendo a distal realizada no nível da bifurcação carotídea (Figura 3).

**Figura 3:** Tomografia computadorizada do pescoço (renderização volumétrica) no 18º mês pós-operatório. * enxerto com jugular externa direita; c: carótida comum direita, as setas apontam as anastomoses proximal e distal do enxerto.

Foi colocado um dreno a vácuo, exteriorizado por contra-abertura, o qual permaneceu por 24 horas. O paciente recebeu alta hospitalar no segundo dia pós-operatório e foi orientado a fazer uso de ácido acetil-salicílico 100 mg/dia durante 3 meses e a manter acompanhamento periódico.

O caso vem sendo acompanhado há 18 meses sem complicações neurológicas. Uma angioTC de controle recente demonstrou perviedade do enxerto venoso, porém com discreta dilatação do calibre ao longo de toda a extensão. Um eco-Doppler colorido demonstrou perviedade do sistema carotídeo, com fluxo laminar na carótida comum pré-anastomose e na carótida interna. Entretanto, devido à dilatação homogênea do enxerto, foi registrado em seu interior um fluxo turbilhonar sem repercussão hemodinâmica. O pico de velocidade sistólica registrado foi de 87,9 cm/s na carótida comum pré-enxerto e 69,2 cm/s na carótida interna.

## DISCUSSÃO

O pescoço é vulnerável a lesões que podem acometer vasos sanguíneos, nervos, traqueia e esôfago^9^. Os traumas penetrantes no pescoço estão associados a lesões vasculares em aproximadamente 25% dos casos^7^ e as lesões carotídeas podem ser potencialmente fatais por hemorragia, compressão da via aérea e acidente vascular cerebral^7^^,^^10^. Fístulas carotídeo-jugulares não são frequentes e correspondem a apenas 4% de todas as FAVs traumáticas^11^.

O diagnóstico da FAV traumática e do PSA requer anamnese e exame clínico cuidadosos; métodos diagnósticos não invasivos e invasivos podem ser necessários^4^^,^^12^. Sinais e sintomas de FAV incluem pulsação na região do pescoço, edema, sopro sistólico, frêmito e dilatação de veias superficiais^12^^-^14. O sopro em maquinaria ou locomotiva é patognomônico de FAV^15^ e o sinal de Nicoladoni-Branham, que se caracteriza por bradicardia e aumento da pressão arterial média quando se realiza a compressão manual da fístula, pode ser observado em alguns casos^16^^-^18.

Em casos de lesão vascular evidente e instabilidade hemodinâmica, é indicada uma intervenção cirúrgica imediata^8^. Tratamentos eletivos devem ser precedidos de planejamento terapêutico criterioso, que requer avaliação anatômica das estruturas acometidas^4^^,^^12^. A angioTC, a ressonância magnética (RM) e a ultrassonografia com Doppler são frequentemente utilizadas; em casos selecionados, a angiografia pode desempenhar um papel importante nesse planejamento, tanto para cirurgias convencionais quanto endovasculares ou híbridas^4^^,^^16^^,^^18^. Nesse caso, a arteriografia mostrava opacificação intracraniana apenas quando o cateter era avançado distalmente à FAV; quando em posição proximal, todo o fluxo era desviado para o sistema venoso pela FAV, demonstrando que, do ponto vista funcional, equivalia à ligadura da carótida, por isso a reconstrução cirúrgica pôde ser realizada sem *shunt* vascular.

Em situações de distorção anatômica, como nas grandes FAVs ou naquelas associadas a PSA, técnicas endovasculares apresentam vantagens sobre a dissecção cirúrgica das estruturas envolvidas^7^^,^^12^. A escolha da técnica deve levar em conta características anatômicas, tamanho do saco aneurismático, grau de lesão arterial e fluxo arterial distal^7^^,^^14^. Além disso, a disponibilidade de materiais, a faixa etária do paciente e as condições de acompanhamento a longo prazo também devem ser consideradas.

Técnicas endovasculares são frequentemente indicadas em lesões da artéria vertebral e envolvendo as carótidas nas zonas I ou III, onde o acesso cirúrgico é mais difícil^7^^,^^18^. As vantagens dessa abordagem são acesso remoto menos invasivo, menor hemorragia, tempo de internação mais curto e consequentemente menor custo^7^^,^^12^. Entretanto, estudos demonstram que o tratamento endovascular de FAV e PSA traumáticos apresenta riscos potenciais de estenose tardia intra-stent, tromboembolismo e formação de PSA no local de punção arterial^4^^,^^12^.

Em relação à técnica cirúrgica convencional, o reparo aberto da fístula carotídeo-jugular pode ser realizado com enxertos, anastomoses termino-terminais, remendos (*patches*), suturas e ligaduras^8^; quando possível a ligadura deve ser evitada, já que está associada a resultados menos favoráveis que a reconstrução arterial^9^. Além disso, a reconstrução arterial com veia autóloga representa uma solução mais durável e com risco mais baixo de infecção em relação ao uso de materiais protéticos^3^^,^^4^. Porém, o enxerto venoso pode apresentar dilatação tardia, especialmente em jovens, como no caso apresentado, o que requer acompanhamento^2^^,^^3^.

A veia safena magna pode ser utilizada com baixas taxas de trombose e infecção, porém requer incisão adicional no membro inferior, com consequente aumento do tempo cirúrgico^2^^,^^8^. Tendo em vista o benefício de um enxerto autólogo e o fato da veia jugular externa já apresentar-se dilatada e com parede espessada (pela pressurização da FAV), a equipe cirúrgica optou pelo uso dela, descartando a necessidade de incisões adicionais e diminuindo o tempo cirúrgico.

O tratamento endovascular estava disponível e o implante de um stent revestido seria factível e tecnicamente mais simples do que a dissecção cirúrgica em um caso como este. Entretanto, a escolha da técnica convencional em detrimento da endovascular levou em consideração os riscos de complicações tardias e a escassez de estudos que avaliem sua eficácia a longo prazo, já que o paciente era muito jovem^2^. Além disso, o reparo endovascular também requer terapia antiplaquetária pós-operatória de longa duração para evitar complicações troboembólicas e oclusão do stent e a vigilância com exames de imagem periódicos, o que dificulta a adesão ao tratamento, especialmente quando o paciente apresenta situação de vulnerabilidade socioeconômica^4^^,^^19^, como é o caso do paciente descrito.
